# Liquor Bismuthi—Preparation

**Published:** 1873-12

**Authors:** C. Mehu


Preparation of Liquor Bismuthi.—*By C. Mehu.*—I dissolve an ascertained weight of pure bismuth in three times its weight of pure nitric acid, then concentrate the solution and leave it to crystalize. After one or two days, the mother liquor which surrounds the crystals is decanted and evaporated in a porcelain capsule at a moderate temperature, so as to completely drive off the excess of acid; in cooling, the liquor forms a crystaline mass. All the crystals being put together, I then pour upon them a concentrated solution of citric acid, made with heat. For each equivalent of bismuth I employ an equivalent of crystalized citric acid, being very nearly equal weights of each. The solution of citric acid dissolves completely the crystals of nitrate of bismuth.

In order to obtain citrate of bismuth I divide this solution of nitrate of bismuth in citric acid into two equal parts, and pour into one of them a sufficient quantity of ammonia to dissolve the precipitate that is formed at first, leaving only a slight excess of ammonia, and then add the other portion of the solution. From this mixture there results a very white precipitate of citrate of bismuth, which I wash with warm water as long as it gives any traces of acidity, and then dry in a stove. The washings are acid and contain a large proportion of nitrate of ammonium, with scarcely any traces of bismuth. This can be isolated in a state of sulphide by means of sulphide of sodium.

The citrate of bismuth so prepared dissolves in ammonia; the solution can be diluted at will with water without becoming turbid, and may be preserved for years.—*London Pharm. Jour.*

